# The Pathway Coexpression Network: Revealing pathway relationships

**DOI:** 10.1371/journal.pcbi.1006042

**Published:** 2018-03-19

**Authors:** Yered Pita-Juárez, Gabriel Altschuler, Sokratis Kariotis, Wenbin Wei, Katjuša Koler, Claire Green, Rudolph E. Tanzi, Winston Hide

**Affiliations:** 1 Department of Biostatistics, Harvard T.H. Chan School of Public Health, Boston, United States of America; 2 Sheffield Institute for Translational Neuroscience, Department of Neuroscience, University of Sheffield, Sheffield, United Kingdom; 3 Genetics and Aging Research Unit, MassGeneral Institute for Neurodegenerative Disease, Massachusetts General Hospital and Harvard Medical School, Charlestown, Massachusetts, United States of America; 4 Harvard Stem Cell Institute, Cambridge, Massachusetts, United States of America; 5 National Institute Health Research, Sheffield Biomedical Research Centre, Sheffield, United Kingdom; Memorial Sloan-Kettering Cancer Center, UNITED STATES

## Abstract

A goal of genomics is to understand the relationships between biological processes. Pathways contribute to functional interplay within biological processes through complex but poorly understood interactions. However, limited functional references for global pathway relationships exist. Pathways from databases such as KEGG and Reactome provide discrete annotations of biological processes. Their relationships are currently either inferred from gene set enrichment within specific experiments, or by simple overlap, linking pathway annotations that have genes in common. Here, we provide a unifying interpretation of functional interaction between pathways by systematically quantifying coexpression between 1,330 canonical pathways from the Molecular Signatures Database (MSigDB) to establish the Pathway Coexpression Network (PCxN). We estimated the correlation between canonical pathways valid in a broad context using a curated collection of 3,207 microarrays from 72 normal human tissues. PCxN accounts for shared genes between annotations to estimate significant correlations between pathways with related functions rather than with similar annotations. We demonstrate that PCxN provides novel insight into mechanisms of complex diseases using an Alzheimer’s Disease (AD) case study. PCxN retrieved pathways significantly correlated with an expert curated AD gene list. These pathways have known associations with AD and were significantly enriched for genes independently associated with AD. As a further step, we show how PCxN complements the results of gene set enrichment methods by revealing relationships between enriched pathways, and by identifying additional highly correlated pathways. PCxN revealed that correlated pathways from an AD expression profiling study include functional clusters involved in cell adhesion and oxidative stress. PCxN provides expanded connections to pathways from the extracellular matrix. PCxN provides a powerful new framework for interrogation of global pathway relationships. Comprehensive exploration of PCxN can be performed at http://pcxn.org/.

## Author summary

Genes do not function alone, but interact within pathways to carry out specific biological processes. Pathways, in turn, interact at a higher level to affect major cellular activities such as motility, growth and development. We present a pathway coexpression network (PCxN) that systematically maps and quantifies these high-level interactions and establishes a unifying reference for pathway relationships. The method uses 3,207 human microarrays from 72 normal human tissues and 1,330 of the most well established pathway annotations to describe global relationships between pathways. PCxN accounts for shared genes to estimate correlations between pathways with related functions rather than with redundant pathway definitions. PCxN can be used to discover and explore pathways correlated with a pathway of interest. We applied PCxN to identify key processes related to Alzheimer’s disease (AD), interpreting a mixed genetic association and experimental derived set of disease genes in the context of gene co-expression. We expand the known relationships between pathways identified by gene set enrichment analysis in brain tissues affected with AD. PCxN provides a high-level overview of pathway relationships. PCxN is available as a webtool at http://pcxn.org/, and as a Bioconductor package at http://bioconductor.org/packages/pcxn/.

## Introduction

The advancement of high throughput, high dimensional ‘omic’ technology has enabled quantification of a vast array of cellular components. Inducing phenotypic changes, through mutations or perturbations, and observing their impact on genomic, proteomic and metabolomic assays has allowed us to assign roles to sets of genes and gene products [1–3]. We now appreciate that cell states are controlled by cascades of interactions coordinated into protein complexes and pathways [4–6]. Thus pathways have become the functional building blocks on which we base interpretation of cell state. However, systems approaches to interpret the relationships between omic components have focused upon development of gene based interrogation through gene-gene networks. Pathways drive biological processes through complex and poorly understood interactions, and only limited functional references for global pathway relationships exist. Mapping out pathway relationships is a fundamental challenge as we strive to influence cell development and disease [7, 8].

### Pathway analysis

The development of databases such as KEGG [9], Reactome [9, 10] and Biocarta [11] have provided curated lists of pathway membership. These gene lists enable systematic mapping of genomic scale data to biological processes. Gene expression profiling provides the most common basis for describing experimental changes in pathway terms. Usually, differentially expressed genes between a pair of conditions are used to highlight enriched pathways. Well established methods such as GSEA [12], SAFE [13], PAGE [14] and GSA [14, 15] produce lists of pathways that are significantly enriched in an individual experiment [16, 17]. A characteristic of these approaches is that pathways are analyzed independently, the co-enrichment of other pathways considered only insofar as necessitating multiple hypothesis testing. Significant gene membership overlap exists between pathways; and similar but not identical names exist for equivalent, but differently constituted, pathways in separate databases. Describing the relationships between pathways with redundant annotations from different sources might capture high-content similarity rather than truly related biological mechanisms [18, 19]. In hierarchical database structures such as GO [20], gene sets corresponding to one process may be fully contained within subset of a parent process. The development of multi-set approaches such as GenGO [21], Markov chain ontology analysis (MCOA) [22], model-based gene set analysis (MGSA) [23], and Selection via LASSO Penalized Regression (SLPR) [24] allows joint testing of pathways for enrichment. Multi-set methods alleviate problems relating to overlap and redundancy, and multifunctional, or pleiotropic, genes that play roles in different biological processes [25]. However, pathways are still treated as independent units without accounting for, or determining, expression correlation arising from biological interaction. Co-enrichment of pathways can either be a reflection of closely related functions or a consequence of overlapping annotation. Pathways also operate in networks, and so pathway-pathway relationships affect their constituent gene expression signatures.

### Pathway networks

A natural extension to gene-centric analysis is to consider the interactions between biological pathways, taking into account relationships between higher level systemic functions of the cell and the organism [26, 27]. The key to existing approaches for mapping pathway relationships has been recognition that genes and their products interact with each other, resulting in combinations of gene network relationships, annotation, functional or semantic classification overlaps [28, 29], protein interactions, and gene and network enrichment [30–35].

### Networks based on annotation

Several methods for connecting pathways rely solely on annotation, using gene overlap to describe the relationships between gene sets. Methods such as Onto-Express [36] and BiNGO [37] use Gene Ontology (GO) [20] as their only source of curated gene sets and identify parent-child relationships of GO gene sets of interest via gene overlap. Since these methods were developed specifically for GO annotations, their applicability is limited to functional annotation within this hierarchical structure. More recent annotation-based methods such as the Molecular Concepts Maps (MCM) [38], the Enrichment Map [39, 40] and the Constellation Map [41] are not restricted to GO. These methods build networks in which the nodes are gene sets and the edge weights are based on shared genes or an intra-experiment similarity score.

### Networks based on curated interactions

Pathway interaction networks can also be defined using distance measures based on aggregating curated gene level connections, such as protein-protein interactions (PPIs) [30, 31, 42, 43] or empirically, based on gene coexpression data [32]. Methods based on PPI such as the pathway crosstalk network (PCN) [43] and the characteristic sub pathway network (CSPN) [42] determine relationships between pathways based on the assumption that two pathways are likely to interact if they share a significant number of PPIs. PCN identifies pathway relationships based on the number of shared interactions from a background PPI network to build a global network of pathway interactions [43]. CSPN identifies pathway interactions for a specific phenotype by counting the number of active PPIs defined from differentially expressed genes and a curated PPI background network [42]. Methods based on PPIs have important limitations; when two pathways share only a few PPIs between them but are still significantly related by other interactions, their functional relationship may be missed by the PPI approach. Moreover, these methods rely heavily on the background network structure, whose comprehensiveness, accuracy and importantly, context, bias the results. Issues with PPIs can be alleviated by integrating additional sources of curated relationships. Network Enrichment Analysis (NEA) [30] and CrossTalkZ [31] use a background gene network that complements PPIs with GO annotations and a network of functional coupling [34] to relate pathways based on the extent of their connectivity.

### Networks based on gene expression

Systems approaches to interpret the relationships between differentially expressed genes have focused upon development of gene coexpression networks, where these genes are related to each other by known coexpression in extensive large scale assays [44, 45]. These methods have been adapted to quantify pathway correlations. For instance, the gene-set coexpression level (GSCoL) method establishes pathway interactions based on sparse canonical correlation analysis of fold change levels derived from gene expression data [32, 34]. The Constellation Map provides an enhanced visualization of GSEA results, by defining a distance between pathway pairs. This distance is based on the per-sample similarity of their enrichments across the experimental data. The similarity is based on normalized mutual information rather than the correlation coefficient to capture nonlinear associations. A limiting issue in these methods is that results are unique to the combination of samples compared, restricting conclusions to a specific context, usually a single experiment. Also, experimental and platform biases can drown out changes in biological signal [46, 47] and complicate cross experiment comparison. Thus far, only limited pathway networks have been constructed and existing approaches are not designed for creating a global reference network that can be used for discovery and mining of pathway relationships. Public omics data archives such as the Gene Expression Omnibus (GEO) [48] and ArrayExpress [49] contain genome-wide gene expression data from a growing number of experiments [50]. These large collections of microarray data allow meta analyses on gene expression that extend the use of thousands of data sets beyond their initial experimental design [51–53]. Harnessing the scope of these repositories is increasingly being realised as a powerful tool for identifying universal genomic features [54–56].

### The Pathway Coexpression Network

In this work, we address the need for a consistent functional map of pathway interactions. A reference network of global relationships between pathways serves two purposes: it allows deeper exploration of basic cell biology, and serves as a tool to discover novel mechanisms and targets in disease while building testable models of pathway interaction. Our aim has been to create a network that delineates the global relationships between canonical pathways in as broad a context as possible. To achieve this goal, we have developed the Pathway Coexpression Network (PCxN). For each experiment from a curated collection of normal human tissue microarrays [54] from publicly available experiments in GEO, we estimated the correlation between pathway summaries based on the mean expression ranks of their gene members along with the corresponding p-value. In the presence of shared genes between the pathway annotations, we adjusted the correlation using the mean expression ranks of the shared genes. Finally, we combined the experiment-level correlation estimates and their corresponding p-values to determine which correlations were significant across all experiments. PCxN significantly expands the scope of pathway methods by estimating global relationships between a wide range of curated pathway annotations, based on coexpression across an expansive gene expression collection. The growing number of available pathway annotations from different sources extends their coverage of biological processes. However, as pathway collections get larger and more complex, the redundancy between the contents of the pathway annotations increases. Pathway coexpression based relationships are often dominated by shared genes. Thus, we have taken into account the shared genes between pathways so the pathway relationships reflect actual related functions rather than similarities in annotations.

Here we report how PCxN effectively captures intra-pathway relationships within known pathways such as the ribosome pathway. Then, we show how PCxN finds pathways associated with a complex disease: Alzheimer’s disease (AD). PCxN determines well known pathways related to AD, including those that influence amyloid pathology and innate immune response. Finally, we show how use of PCxN can complement and expand the results of gene set enrichment analysis within an AD gene expression profiling study. PCxN helps to interpret the results by describing the relationships between the enriched pathways, and provides the opportunity to discover novel relationships by revealing pathways which are highly correlated with the enrichment results. PCxN addresses the need to describe relationships between pathways present across diverse tissues and conditions. These relationships provide a pathway interaction model for a biologically driven phenotype, provide a reference to prioritize targets of biological processes, and provide a powerful enhancement for interpretation of results from gene set enrichment methods. We have built a comprehensive web tool for PCxN to explore novel relationships and to aid with the interpretation of results from gene set enrichment methods (http://pcxn.org/). In addition, PCxN is available as a Bioconductor package (http://bioconductor.org/packages/pcxn/).

## Results

### PCxN overview

**Fig 1:**
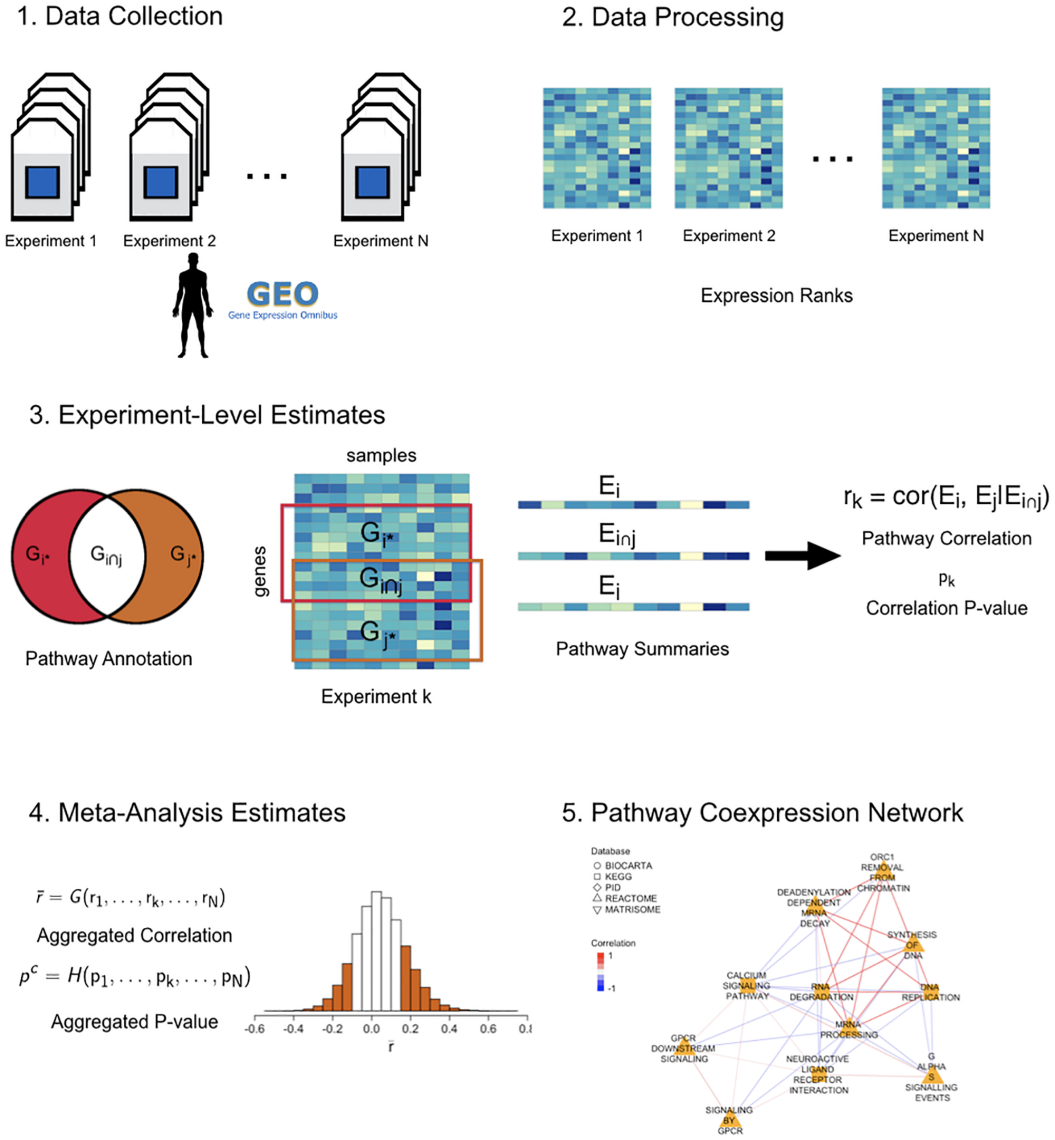
Pathway Coexpression Network (PCxN) overview. (1) Human gene expression arrays for normal human tissues curated from GEO in Barcode 3.0 (2) The gene expression levels were replaced by their ranks so all arrays share a common scale. (3) For each microarray experiment, we first estimated the pathway expression based on the mean of the expression ranks, then the pathway correlation adjusted for shared genes, and tested the significance of the correlation. (4) We aggregated the experiment-level estimates to get the global pathway correlation and its corresponding significance. (5) We built a pathway coexpression network based on the significant pathway correlations.

PCxN is a weighted undirected network in which the nodes represent pathways and the edges are based on the correlation between the expression of the pathways. We built PCxN using 1,330 pathways from the Molecular Signatures Database (MSigDB v.5.1) [57] and 3,207 human microarrays from 72 normal human tissues from GEO curated in Barcode 3.0 [48, 54, 57]. The network was created by first ranking normalized gene expression levels to provide a uniform scale for all samples, an approach similar to the Pathprint method [56]. Ranks provide robust summary statistics to calculate expression scores that do not depend on the dynamic range of an array [58, 59]. Pathways were assigned an expression summary in each array based on the mean rank of its constituent genes. Since our gene expression background is composed of several experiments representing different tissues, for each pair of canonical pathways we estimated the correlation between their expression summaries and tested for significance in every experiment. Then we combined the experiment-level estimates into global estimates. Two pathways are connected in the coexpression network if the correlation coefficient between them is significant after adjusting for multiple comparison. Our goal is to describe the relationships between canonical pathways when their functions are related, rather than when their annotations have similar content. The pathway correlations in the network were adjusted to account for the shared genes between pathway pairs. If a pathway pair shares genes, we estimate the correlation between the pathway summaries conditioned on the summary for the shared genes (Fig 1).

#### Significant correlations within the ribosome pathway

To determine how effectively PCxN captures tightly related biological functions we analysed the ribosome pathway (KEGG accession hsa03010). The KEGG *Ribosome* pathway is a gene set that represents a well characterized, meaningful and ubiquitous biological function [60–63]. We compared the pathway correlation coefficients and the corresponding p-values estimates from permuted gene sets generated from within the ribosome pathway with estimates from random gene sets. Since our method accounts for the contribution of shared genes to estimate the pathway correlation, we considered cases where the gene sets shared no genes, and cases with different degrees of gene overlap. In the no overlap case, we created ribosome gene sets by permuting the genes in the ribosome pathway (126 genes) and splitting them into two separate gene sets. The corresponding random gene sets were created by sampling 126 genes at random and splitting them into two. For the overlap cases, the gene sets were split into two gene sets sharing genes. We used the overlap coefficient to describe the overlap between gene sets represented as pathways. The overlap coefficient between two sets is the size of the intersection divided by the size of the smaller of the two sets. Unlike other measures of set overlap, the overlap coefficient between two sets is always 1 whenever one of the sets is a subset of the other, and always 0 whenever the two sets are disjoint. A key feature of PCxN is to estimate the correlation between gene sets taking into account their shared genes, so we decided to use the overlap coefficient to describe the degree of overlap between the pathway annotations. We considered 9 different overlap cases, ranging from low overlap (overlap coefficient *o*_*AB*_ = 0.0469) to high overlap (overlap coefficient *o*_*AB*_ = 0.8532).

**Fig 2:**
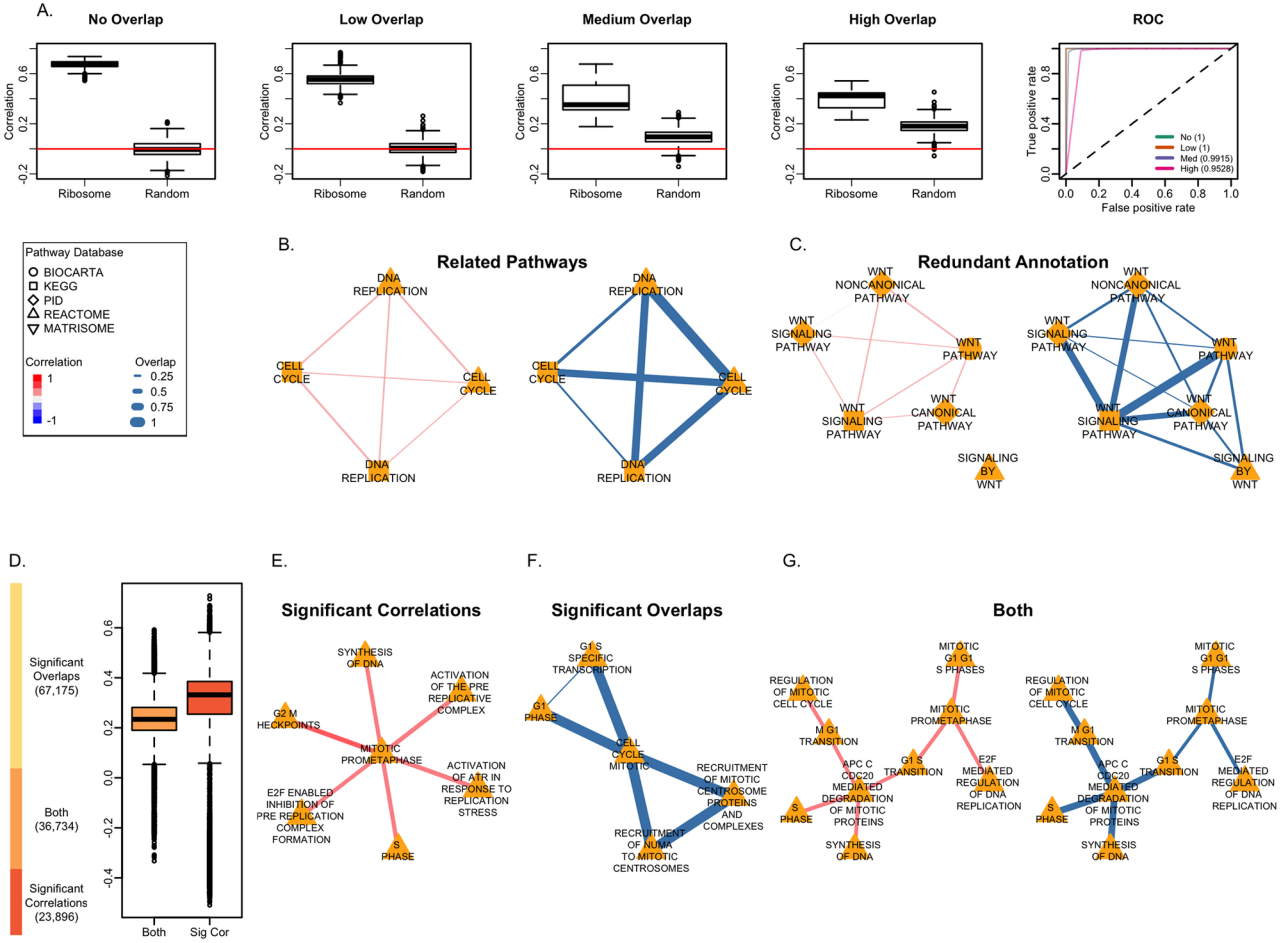
Significant correlations between the ribosome pathway and impact of gene overlap. (A) Boxplots of the correlation estimates between the Ribosome gene sets and random gene sets, and receiver operating characteristic (ROC) curves with the corresponding area under the curve (AUC) values in parenthesis under different degrees of overlap: no overlap, low overlap (overlap coefficient 0.0469, AUC = 1), medium overlap (overlap coefficient 0.5517, AUC = 0.9915) and high overlap (overlap coefficient 0.8532, AUC = 0.9528). The shape of the node in the following networks corresponds to the pathway database. For coexpression networks, the edge color indicates the value of the correlation and edge width is proportional to the correlation magnitude. For the overlap networks, the edge width is proportional to the overlap coefficient. (B) Pathway coexpression and overlap network for the KEGG and Reactome annotations of the *Cell Cycle* and *DNA Replication* pathways. These pathways have related functions and share genes between them. (C) Pathway coexpression network and overlap network for different versions of the *Wnt Signaling* pathway. In the coexpression network, missing edges correspond to correlations that are not significant. These pathway annotations are redundant and represent the same function (D) The stacked bar plot shows the number of pathways pairs with only significant correlations in red, with only significant overlaps in yellow, and with both in orange. The boxplots show the distribution of the correlation coefficients with pathway pairs with only significant correlations (red) and with both significant overlaps and significant correlations (orange). (E) Pathway coexpression network for the Reactome pathways related to the mitotic metaphase of the cell cycle with significant correlations but no shared genes. (F) Overlap network for Reactome pathways related to the mitotic cell cycle with significant overlaps but no significant correlations. (G) Pathway coexpression network and overlap network for cell cycle phases and related processes from Reactome with both significant correlations and significant overlaps.

The correlation estimates from the ribosome gene sets are positive while the estimates for the random gene sets are smaller in magnitude and closer to zero (Fig 2A). Under the assumption that a significant p-value for ribosome gene sets is a true positive while a significant p-value for random gene sets is a false positive, we assessed the ability of our method to identify truly significant correlation coefficients. All the p-values from the ribosome gene sets were significant, while most of the p-values for the random gene sets were not significant. This trend is evident in the receiver operating characteristic (ROC) curves for the no overlap and overlap cases (Fig 2A).

### Accounting for gene overlap

Pathway annotations from different sources present challenges when relating pathways: equivalent pathways with different annotations have similar but not identical names, annotations exist for equivalent but differently constituted pathways in separate databases, and pathways with completely different names share genes [18, 19]. The MSigDB canonical pathways collection is a curated selection of pathway annotations from other databases: Reactome [64], KEGG [65], the Pathway Interaction Database (PID) [66], Biocarta [11], and the Matrisome Project [67].

#### PCxN and redundant pathways

An example of pathway annotation redundancy within MSigDB includes annotations from Reactome and KEGG for both the *Cell Cycle* and the *DNA Replication* pathways (Fig 2B). These pathways share genes between each other because they represent the same processes, and DNA replication is a function related to the cell cycle. In the Reactome annotations, the *DNA Replication* pathway is a subset of the *Cell Cycle* pathway. The pathway correlation is significant and positive for these pathways. In other cases, there is more than one annotation for the same pathway. MSigDB has annotations from KEGG, Biocarta, Reactome and the Pathway Interaction Database (PID) for the *Wnt signaling* pathway. These annotations share genes among each other. Unlike the previous example, the correlation estimates between the Wnt signaling pathways have a small magnitude and most of them are not significant (Fig 2C). Our motivation to account for shared genes between pathways is to assign significant correlation coefficients between pathways representing related functions and non-significant correlation coefficients for pathways with redundant annotations representing the same function.

#### Impact of gene overlap

In order to understand the trade-offs resulting from discarding shared genes in estimating the correlation in PCxN, we compared significantly correlated pathways with pathways where the amount of shared genes is significant according to Fisher’s exact test. We decided to use Fisher’s exact test because this test has been widely used to describe relationships between gene sets based on shared genes in methods such as POSOC [68], Ontologizer [69], GOstats [70]. Furthermore, the Molecular Concepts Map (MCM) [71] uses Fisher’s exact test as similarity score between gene sets to build networks for gene sets. Of all canonical pathway pairs, 19% have only significant correlation coefficients, 52% have only significant overlaps and 29% have both (Fig 2D).

PCxN has an advantage over overlap based approaches when we consider pathways with related functions but without shared genes. For example considering the Reactome pathways, the *Mitotic Prometaphase* pathway describes a function related to the cell cycle, is significantly correlated with other Reactome pathways involved in cell cycle, but does not have genes in common with them (Fig 2E). On the other hand, the correlation from PCxN is not significant between pathways with a very high gene overlap even though these pathways might represent closely related functions. For instance, pathway annotations from Reactome representing different aspects of the mitotic cell cycle as well as other closely related cell cycle processes have a significant gene overlap with the general *Cell Cycle Mitotic* pathway but are not significantly correlated (Fig 2F). However, some pathways with related functions have both significant correlations and significant overlaps. For instance, we identified Reactome pathways for mitotic cell cycle phases and related processes that are significantly correlated and have significant overlap among them (Fig 2G). The *APC/C CDC20 Mediated Degradation of Mitotic Proteins* pathway is both significantly correlated and has significant overlaps with the *Synthesis of DNA*, *S Phase*, *M/G1 Transition* and *G1/S Transition* pathways. The ubiquitin ligase anaphase-promoting complex or cyclosome (APC/C) initiates chromatid separation and entrance into anaphase [72], and the cell-division cycle protein 20 (CDC20) is an essential regulator of cell division that activates APC/C [73, 74]. The *E2F Mediated Regulation of DNA Replication* pathway is significantly correlated and has a significant overlap with the *Mitotic Prometaphase* pathway which in turn is significantly correlated and has a significant overlap with the *G1/S Transition* pathway. The E2F family of transcription factors play a major role during the G1/S transition in mammalian and plant cell cycle [75].

### Case study: Alzheimer’s disease (AD)

With the goal of determining the value of our approach in understanding pathway relationships in complex disease, we chose an important disease for which there is abundant transcriptomic data, established genetic associations, and the need for better understanding of the roles of pathways and their relationships is fundamental to the prioritisation of drugs and drug targets. AD is a progressive multifarious neurodegenerative disorder [76, 77] and the most common type of dementia. AD is one of the great health-care challenges of the 21st century [78]. Pathologically it is characterized by intracellular neurofibrillary tangles and extracellular amyloidal protein deposits contributing to senile plaques [76]. While the neuropathological features of AD are recognized, little is known about the causes of the disease and no curative treatments are available [76, 78]. We chose this disease to illustrate how the PCxN can reveal important or even novel functional relationships underlying a complex pathological phenotype. We performed a series of additional analyses that bring together genes that have been identified by totally independent assays: genetic and transcriptomic surveys associated with AD.

**Fig 3:**
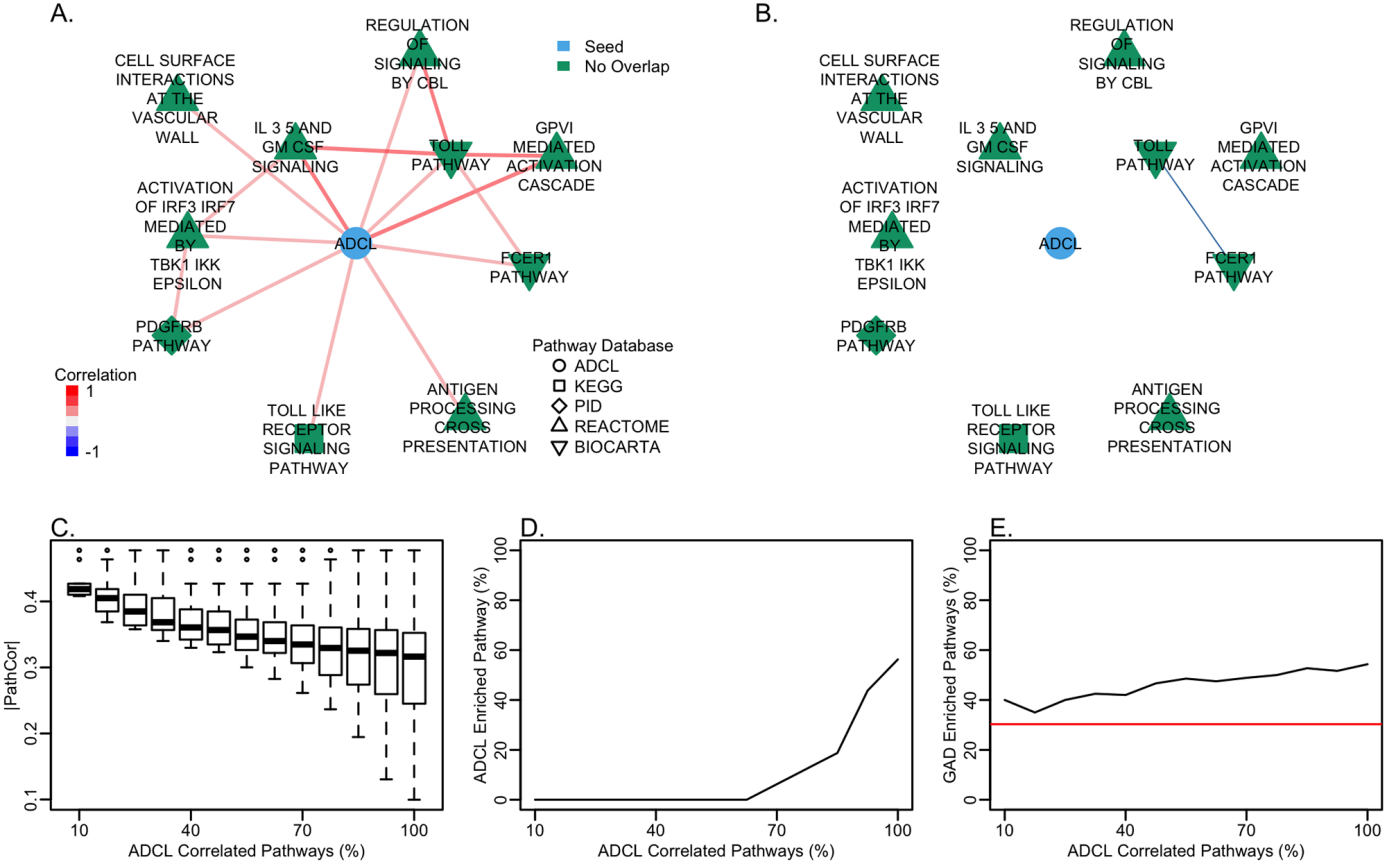
Canonical pathways correlated with the Alzheimer’s disease curated list. The ADCL is colored in blue. Neighbors without genes in common with the ADCL are highlighted in green. The shape of the node corresponds to the pathway database. For the coexpression network, the edge color indicates the value of the correlation and the edge width is proportional to the correlation magnitude. For the overlap network, the edge width is proportional to the overlap coefficient. (A) Pathway coexpression network for the top pathways correlated with the ADCL (by correlation magnitude). All correlated pathways have established associations with AD: *GPVI Mediated Activation Cascade* [79], IL-3, 5 and GM-CSF signalling [80], *Antigen Processing Cross Presentation* [81], *PDGFRB Pathway* [83], *Toll Pathway* [84], *Regulation of Signaling by CBL* [82], *Toll-like Receptor Signaling* [85], *Activation of IRF3/IRF7 Mediated by TBK1/IKK Epsilon* [85], *Cell Surface Interactions at the Vascular Wall* [86], *FCER1 Pathway* [87]. (B) Shared genes (overlap coefficient) between the top pathways correlated with the ADCL. (C) Correlation magnitude of all canonical pathways correlated with the ADCL sorted by the magnitude of their correlation and split in bins of increasing size. (D) Proportion of canonical pathways enriched for the genes within the ADCL (*p* < 0.001, adjusted with FDR) present in the canonical pathways correlated with the ADCL (E) Proportion of canonical pathways enriched for genes associated with AD from the Genetic Association Database present in the pathways correlated with the ADCL (*p* < 0.001, adjusted with FDR). The red line indicates the proportion of all 1,330 canonical pathways enriched for genes within the ADCL.

We used genes within an AD curated list (ADCL) as the disease gene signature. The ADCL is a set of association-derived and experimental-derived genes related to AD. Consisting of 68 genes of which 61 genes were present in the PCxN gene expression background (S4 Table). The ADCL is the result of expert assessment of the current understanding of AD from a combination of key genes from genome-wide association studies and from functional analyses. We integrated the ADCL to PCxN first by estimating all the pairwise correlations between the summary for its constituent genes and the summaries for the canonical pathways adjusted for overlap across each experiment in the gene expression background along with the corresponding p-values. Then, we aggregated the experiment level correlation estimates and combined the p-values. Finally, we adjusted the combined p-values from the correlations with the ADCL with the rest of the combined p-values from the correlations between the canonical pathways for multiple comparison using FDR. PCxN allowed us to identify canonical pathways significantly correlated with the curated AD gene list. The top 10 correlated pathways (Fig 3A) are all known to be related to Alzheimer’s disease or amyloid pathology [79–87] and the majority of the top 25 correlated pathways (S5 Table) are related to immune responses. The top correlated pathway to ADCL, *GPVI Mediated Activation Cascade*, is associated with regulation of Amyloid beta (A*β*). GPVI and FCER1 initiate platelet activation that leads to activation of Syk. Syk enhances the formation of stress granules that are prevalent in AD affected brains. The stress granules produce reactive oxygen and nitrogen species that are toxic to neuronal cells. Downregulation of Syk expression reduces A*β* production and increases the clearance of A*β* across the blood-brain barrier [79]. Since PCxN does not rely on shared genes, PCxN uncovers relationships that would have been missed by methods that rely only on gene overlap to describe the relationships between pathways. All of the top ten correlated pathways (Fig 3B) have no genes in common with the ADCL (S5 Table).

To explore novel insights resulting from the use of PCxN, and as a complement to enrichment methods based on gene overlap, we compared the top ADCL correlated pathways with pathways significantly enriched for genes in the ADCL. First, we ordered all pathways correlated with the ADCL (S5 Table) by the magnitude of their correlation and split the pathways into bins of increasing size (Fig 3C). We began with a bin including the 10 most correlated pathways. Every following bin includes 10 additional correlated pathways, so the last bin contains all pathways correlated with the ADCL. For each bin, we calculated the proportion of pathways significantly enriched for the ADCL. As we move across bins, the proportion of ADCL enriched pathways increases (Fig 3D). Furthermore, none of the top 30 correlated pathways was enriched for genes in the ADCL.

#### Enrichment for AD associated genes in ADCL correlated pathways

To assess the validity of the ADCL correlation results, we tested the enrichment of genes associated with AD in pathways correlated with the ADCL using independent methods [88, 89]. We assessed relationships using genetic association by retrieving genes inferred to be associated with AD from the Genetic Association Database (updated August 18, 2014). The Genetic Association Database (GAD) is a comprehensive archive of published genetic association studies that provides a repository of genetic association by data aggregation from genome-wide association and other genetic association studies [88]. We retrieved 668 genes associated with Alzheimer’s disease of which 534 are present in the gene expression data from GEO (S6 Table). We used Fisher’s exact test to determine which of the canonical pathways in PCxN correlated with the ADCL are significantly enriched for genes associated with Alzheimer’s. The ADCL has 14 genes in common with genes associated with Alzheimer’s in GAD, and the overlap is highly significant (*p* = 7.34 × 10^−9^).

Of the top 10 pathways correlated with the ADCL, 6 out of 10 were significantly enriched with genes related to Alzheimer’s found by genetic association. We sorted the ADCL neighbors by the magnitude of their correlation with the ADCL and split them into bins of increasing size (Fig 3C). As we move across the bins, the proportion of pathways significantly enriched for genes related to Alzheimer’s in the neighbors of the Alzheimer’s curated list was higher compared to all of canonical pathways; out of 1330 canonical pathways, 403 (30%) were significantly enriched after adjusting for multiple comparison using FDR and p-value cut-off of 0.001 (S7 Table, Fig 3E). The enrichment results demonstrate a significant link between the correlation of pathways with curated AD genes and genes found independently by genetic association with Alzheimer’s.

### Complement to GSEA: Revealing relationships between enriched pathways

PCxN can be used effectively to determine relationships between pathways as a complement to interpret gene set enrichment (GSE) methods. A typical GSE result is a list of gene sets that are significantly enriched by a list of query genes. PCxN can describe the relationships between the enriched gene sets using the global pathway correlation estimates. To explore correlation between gene sets enriched with a set of query genes, we used Gene Set Enrichment Analysis (GSEA) [12] to find pathways from the MSigDB canonical pathways collection enriched for genes differentially expressed in an AD expression dataset (GSE5281) consisting of genes expressed in post mortem samples of AD in the superior frontal gyrus (S8 Table). The expression data set consisted of 34 superior frontal gyrus samples: 11 controls (clinically and histopathologically normal aged human brains) and 23 affected with AD [90] (S9 Table).

**Fig 4:**
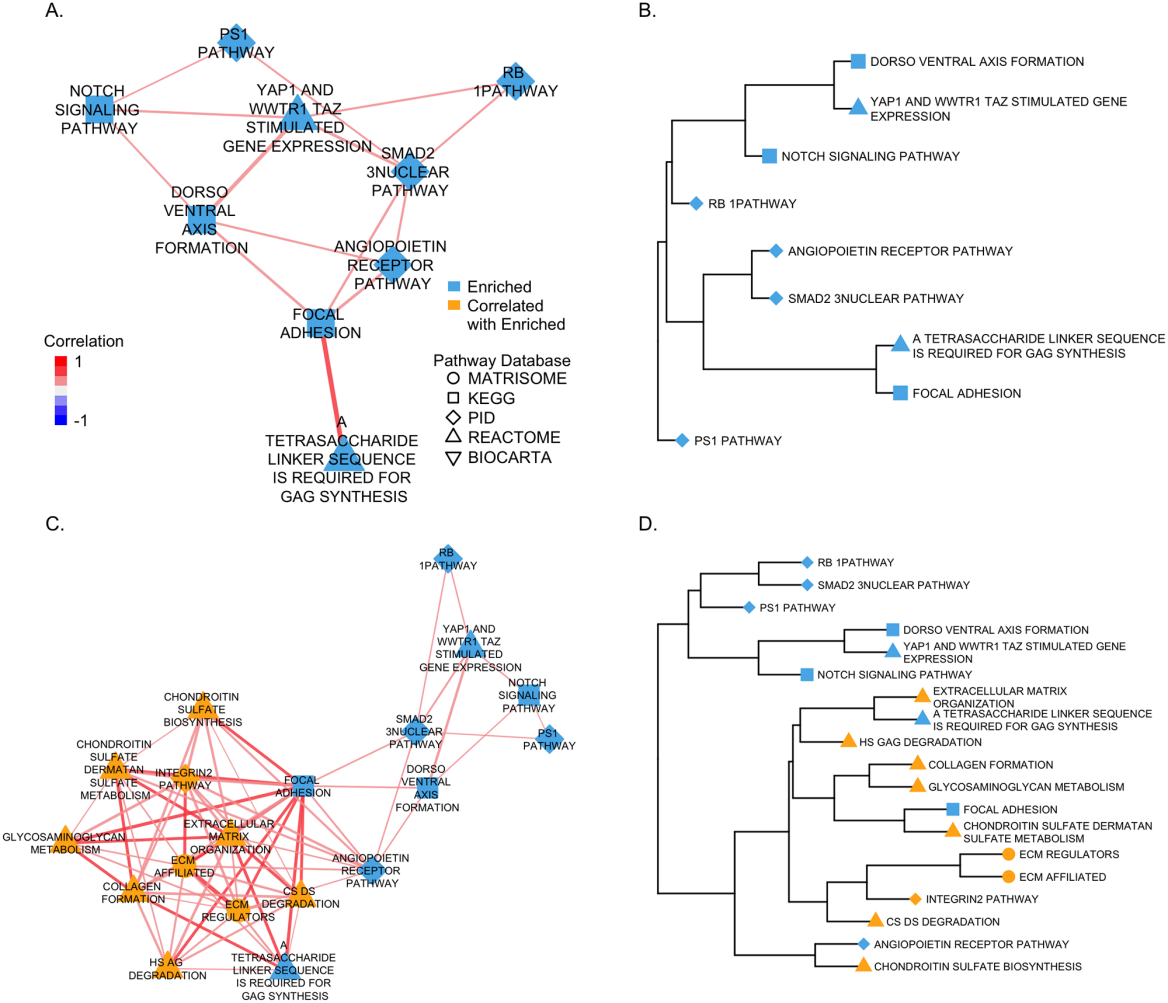
Pathway coexpression for GSEA enriched canonical pathways. GSEA enriched pathways are colored in blue, correlated pathways are yellow. The shape of the node corresponds to the pathway database, the edge color indicates the value of the correlation and the edge width is proportional to the correlation magnitude. (A) Pathway coexpression network for the top 10 GSEA enriched canonical pathways. (B) Hierarchical clustering using average linkage and 1 − |PathCor| as the distance between the top 10 GSEA enriched canonical pathways. (C) Pathway coexpression network for the GSEA enriched pathways and their top 10 correlated pathways (by |PathCor|). (D) Hierarchical clustering using average linkage and 1 − |PathCor| as the distance between the top 10 GSEA enriched canonical pathways and their top 10 correlated pathways.

We chose to examine the functional relationships among the top ten enriched pathways identified by GSEA. Functionally, they all appear to be consistently associated with the AD literature (e.g. the *PS1 Pathway* role in AD [91]). We retrieved significant correlations between the enriched pathways to explore their functional relationships as revealed by PCxN (Fig 4A). To explore the most closely functionally related pathways, we clustered the enriched pathways based on their correlations (Fig 4B). The cluster containing the highest correlations consists of pathways involved in cell adhesion and oxidative stress response (*Focal Adhesion*, *A Tetrasaccharide Linker Sequence is Required for GAG Synthesis*, *Angiopoietin Receptor* and *SMAD2/3 Nuclear Pathway* (S10 Table)). These pathways shared reported functions. Focal adhesions have been implicated in regulating A*β* signalling and cell death in AD [92]. As part of cell adherence to the extracellular matrix (ECM), integrins are activated and the focal adhesion pathway is activated. The ECM/integrin/focal adhesion pathway is involved in the regulation of anchorage-dependent cell survival. Cell adhesion to ECM and overexpressing FAK (focal adhesion kinase), member of *Focal Adhesion Pathway*, is protective against oxidative stress, which has been observed in AD brains [93]. FAK also has the ability to regulate several other cell-death or survival pathways [92]. Members of *A Tetrasaccharide Linker Sequence is Required for GAG Synthesis* are also involved in cell adhesion, which plays an important role in cell death/survival. Members of this pathway include neurocan and brevican, whose expression is mostly restricted to neuronal tissues [94]. Loss of brevican is associated with loss of synapses [95], while A*β* has been shown to increase neurocan expression in astrocytes [96]. In addition to adhesion molecules, angiopoietins (members of the clustered *Angiopoietin Receptor Pathway*) share function as they are activated in response to oxidative stress. Elevated Angiopoietin-1 serum levels can be observed in patients with AD [97]. The closely clustered *SMAD2/3 Nuclear Pathway* contains SMAD3 which regulates expression of angiogenic molecules in tumor cells and vascularization in tumor lesions [98]. SMADs transduce extracellular signals from Transforming Growth Factor *β* (TGF*β*) to the nucleus [99]. SMAD3, one of the key members of the SMAD2/3 nuclear pathway, is down regulated in AD [100], while TGF*β* is upregulated. The imbalance between SMAD3 and TGFA*β* signalling, shifts the regulatory signalling towards a dysregulated inflammatory activation potentially leading to neurodegenerative changes, such as decreased A*β* clearing [100].

The other top ten pathways identified in this GSEA have also been associated with AD and some show documented functional relationships. PS1 is well known as a common cause of familial AD [101]. *Dorso-ventral Axis Formation* has been suggested as one of the pathways regulated by miRNAs identified in a bioinformatics study of Drosophila AD models [102]. Notch is coexpressed with PS1 and altered in AD affected brains [103], YAP1 and WWTR1/TAZ mediate gene transcription induced by the A*β* protein precursor and its paralogues [104]. Finally, increased levels of hyperphosphorylated RB protein have been observed in AD [105] indicating that neurons in AD attempt to re-enter the cell cycle [106].

### Complement to GSEA: Expanded enriched gene sets

In addition to providing relationships between the GSEA results, PCxN can provide potentially novel relationships by retrieving canonical pathways significantly correlated with the pathways identified as enriched. We retrieved the top 10 canonical pathways which were the most correlated with the AD GSEA enriched gene sets, and clustered the correlated pathways along with the results from GSEA (Fig 4C–4D). Most of the top correlated neighbors are components of extracellular matrix (ECM) and form a highly-correlated cluster (Fig 4D) with the top correlated GSEA pathways. The ECM components revealed by PCxN have been highly studied in relation to Alzheimer’s [95, 107–110]. The ECM changes significantly during the early stages of AD [111], but only a limited number of individual ECM components have been studied so far [112].

### Exploring PCxN

We created a user-friendly webtool (http://pcxn.org) that can be used to interactively explore and visualise pathway relationships found in PCxN. The tool allows a user to query the various pathway databases using one or more pathways and retrieve correlation estimates, p-values and overlap coefficients. Since the correlations adjusted for shared genes are a complementary perspective to relationships based on gene overlap, the webtool also provides the option to view coexpression networks based on correlation coefficients not adjusted for shared genes in addition to the PCxN coexpression network that is based on the adjusted correlation. The results are presented through heatmaps (which also offer clustering of pathways), interactive networks (with multiple pre-made structures) and data tables. Pathway members are also retrievable along with their descriptions. In addition, PCxN is available as Bioconductor software (http://bioconductor.org/packages/pcxn/) and data (http://bioconductor.org/packages/pcxnData/) packages which contain the same exploratory/visualization functionality and data as the webtool.

## Discussion

We have developed and described PCxN, a coexpression method to describe global relationships between pathways. PCxN estimates the correlation between 1,330 canonical pathways using a curated collection of 3,207 microarrays in 134 experiments from 72 normal human tissues. We integrated a wide range of experiments by estimating the correlation between summaries of the pathway expression, testing their significance in every experiment, and then aggregating the experiment-level estimates into global estimates. We used gene sets derived from permutations of the *Ribosome* pathway (KEGG) and random gene sets to show that PCxN effectively captures relationships between gene sets with related functions while discarding relationships from random gene sets. The correlation estimates between the ribosome gene set were positive and significant, while the correlation estimates for random gene sets were not significant and with a magnitude close to zero. These results suggest that the correlation between two pathways with related functions is significant.

The influence of redundant annotations across pathways databases is often overlooked. Pathway databases often include pathways that share genes with one another to varying degrees. Shared genes between pathways can either be a consequence of closely related functions or redundant annotation from different sources. Ignoring such redundancies during pathway analysis can lead to identifying pathways relationships due to high content-similarity, rather than truly related biological mechanisms. PCxN adjusts the correlation between pathways by conditioning on the shared genes. The correlations between redundant annotations for the *Wnt signaling* pathway had a small magnitude and were mostly not significant. When pathways share genes due to related functions, the correlations between them might be significant depending on the degree of the overlap. For instance, we found pathways for mitotic cell cycle and related processes that were significantly correlated and had significant overlaps between them. The significant correlations and significant overlaps between these pathways revealed known relationships between ADC/C, CDC20 and the E2F family of transcription factors with the mitotic cell cycle. However, the correlations between a different set of pathways representing other aspects of the mitotic cell cycle, such as the *Mitotic Cell Cycle* and the *G1 Phase* pathways and related processes, such as the *Recruitment of Mitotic Centromere Proteins and Complexes*, were not significant while the overlap was highly significant. PCxN was successful in uncovering relationships between the *Mitotic Prometaphase* pathway and other cell cycle related pathways such as the *G2/M Checkpoints* and the *S Phase* that do not have genes in common.

PCxN provides powerful means to generate models for complex diseases by providing pathways significantly correlated with an assay-independent disease gene signature. We used PCxN to identify key processes related to Alzheimer’s disease (AD) using an AD curated list (ADCL). The top pathways correlated with the ADCL have known relationships with AD or amyloid pathology. Furthermore, the correlated pathways were significantly enriched for genes associated with AD independently derived from genome wide association studies. These results show the value of PCxN in finding biological processes associated with complex diseases using gene signatures. PCxN provides a powerful contribution to the interpretation of the gene set enrichment methods by describing the relationships between enriched pathways independent of gene overlap. We used PCxN to describe the relationships between pathways identified as enriched by GSEA in a published microarray gene expression experiment profiling the effect of AD in the superior frontal gyrus. We expanded the scope of gene set enrichment results by retrieving pathways correlated with the enriched pathways. The top pathways correlated with the enriched pathways are components of extracellular matrix (ECM) and form a highly correlated cluster. We note that the ECM undergoes significant changes during the early stages of AD, but only a few ECM components have been studied. The relationships between the ECM pathways from PCxN could provide leads to future studies of the individual ECM components.

PCxN relies on the completeness and correctness of pathway annotations to relate biological processes. Also, PCxN only considers a pathway as a gene list, omitting any knowledge of the interaction between its members. PCxN is also limited by the gene expression data used to estimate the correlations. The current implementation only uses one microarray platform and a curated expression background. It is widely accepted that pathway activation is phenotype dependent. Using the PCxN approach it will be possible to explore whether pathway-pathway relationships change in relationship to a phenotype, or if consistent functional links prevail irrespective of cell state. Further work is required to investigate how network topology changes with expression background, and in particular into whether pathway networks are significantly disrupted in disease. This implementation of PCxN does not take advantage of the growing number of publicly available RNA-seq data. In future, the method will be expanded to include a wider range of pathway annotations and to use gene expression data from other platforms such as RNA-seq.

PCxN establishes the utility of describing relationships between pathways in a broad context. By using a diverse set of gene expression experiments, PCxN leverages correlation estimates across various human tissues effectively capturing relationships regardless of shared genes. We expect that PCxN can serve as a basis for a high-level map of the relationships between biological process. We built an interactive web-tool that provides a user-friendly portal to explore the PCxN at http://pcxn.org/, as well as a Bioconductor software (http://bioconductor.org/packages/pcxn/) and data (http://bioconductor.org/packages/pcxnData/) package.

## Materials and methods

### Data collection

#### Gene expression data retrieval

We used 134 experiments with 3,207 Affymetrix Human Genome U133 Plus 2.0 microarrays from 72 normal human tissues manually curated in Barcode 3.0 [54] (S1 Table). The curated microarrays in Barcode 3.0 were filtered to exclude poor quality samples [54, 113]. We used the R package GEOquery [114] to retrieve raw CEL files from the Gene Expression Omnibus (GEO) [55]. We processed the raw data with fRMA [115]. We obtained the annotation for the array platform from [116]. To resolve redundancies, multiple probes were mapped to unique Entrez Gene IDs by their mean expression level.

#### Pathway annotations

We retrieved the C2: Canonical Pathways collection from MSigDB [12] (v5.1 updated January 2016). The collection is a curated selection of pathway annotations from other databases: Reactome [64], KEGG [65], the Pathway Interaction Database (PID) [66], Biocarta [11], and the Matrisome Project [67] (S2 Table).

### Experiment-level estimates

Since the microarrays from the gene expression background belong to different experiments representing different tissues, pooling the microarrays to estimate the correlation between pathways would ignore the underlying structure of the data. Even if the correlations are homogeneous, pooling the data is not a valid procedure in general. The pooled estimates may be severely biased due to the heterogeneity of the experiments [117, 118]. Instead of pooled estimates, we first estimated the pathway correlation coefficients and their corresponding p-values for each experiment, and then we combined the experiment-level estimates into global estimates.

### Pathway expression

We represent an experiment with *L* samples as the *K* × *L* matrix *X* where *K* is the total number of genes in the array. Thus, the element *x*_*kl*_ of the matrix *X* corresponds to the expression for gene *k* in array *l*. For each array, the genes were ranked by their expression level. Rank normalizations do not depend on the dynamic range of an array and provide a common range. We represent the expression ranks as the *K* × *L* matrix *S*, where *L* is the total number of arrays and *K* is the total number of genes in the array. Since within each array the genes are ranked by expression level, from 1 (low expression) to *K* (high expression), the entries of the matrix *S* are

$$\begin{array}{c} S_{kl}=\underset{1\leq l\leq L}{\mathrm{rank}}\left(x_{kl}\right) \end{array}$$

where *x*_*kl*_ is the expression level for gene *k* in array *l*.

In this approach pathways are represented as gene sets: groups of functionally related genes. Thus, a pathway is represented by its gene set annotation *G* = {*g*_1_, …, *g*_*n*_}. The pathway expression *E* is a gene set summary statistic based on the expression ranks of the pathway genes; the pathway expression *E* is the mean of the expression ranks of the pathway genes. Consider an experiment with *L* samples, the experiment-level summary for pathway *G* is given by the *L* × 1 vector *E* with entries

$$\begin{array}{c} E_{l}=\frac{1}{n}\sum_{g\in G}S_{gl} \end{array}$$

To calculate *E*, first we take the rows from *S* corresponding to the genes {*g*_1_, …, *g*_*n*_} to get the matrix of ranks of the pathway constituent genes, and then we take the mean across the columns of this matrix, producing the *L* × 1 vector *E*.

Compared to other summary statistics, the mean is fast to compute and easy to interpret. We considered several approaches for the pathway summary statistic, but we found that in most cases the mean performed well. For instance, we considered a summary based on principal components analysis (PCA) but the variance explained by the first principal component was less than 50% for all canonical pathways in the majority of the gene expression experiments from the curated collection of normal human tissues (S1 Text).

### Pathway correlation

#### Shrinkage estimator

We used a shrinkage estimator to compute the experiment-level pathway correlation coefficients. In our setting, a shrinkage estimator will give more reliable experiment-level correlation estimates for experiments with few samples and will set correlation coefficients with a small magnitude to 0 [119]. The shrinkage estimator R* is a linear combination of the standard correlation estimator *R* and a restricted submodel of the correlation matrix

$$\begin{array}{c} R^{*}=\lambda T+\left(1-\lambda \right)R \end{array}$$

where 0 ≤ λ ≤ 1, *R* is the empirical correlation matrix and *T* is identity matrix.

The restricted submodel *T* assumes that all of the variables are uncorrelated. The optimal λ is found by minimizing the mean squared error *L*(λ) between the shrinkage estimator R* and the true correlation matrix *P*.

$$\begin{array}{c} L\left(\lambda \right)={\left\|R^{*}-P\right\|}_{F}^{2}=\|\lambda T-\left(1-\lambda \right)R-{P\|}_{F}^{2}=\sum_{i=1}^{p}\sum_{j=1}^{p}{\left(\lambda t_{ij}+\left(1-\lambda \right)r_{ij}-\rho_{ij}\right)}^{2} \end{array}$$

The analytical solution λ* for the optimal λ [120]

$$\begin{array}{c} \lambda^{*}=\underset{\lambda }{\text{argmin}\;}L\left(\lambda \right) \end{array}$$

is guaranteed to exist and minimize the mean squared error *L*(λ). The solution [119] is given by

$$\begin{array}{c} \lambda^{*}=\frac{\sum_{k\neq l}^{}\text{Var}\left(r_{kl}\right)}{\sum_{k\neq l}^{}r_{kl}^{2}} \end{array}$$

#### Gene overlap

Since genes can be involved in more than one biological process and often pathways share genes, we accounted for the gene overlap between pathways to determine the coexpression between two pathways. Our goal is to describe relationships between patwhays representing related functions rather than pathways with similar annotations. For pathway *i* with gene set *G*_*i*_ and pathway *j* with gene set *G*_*j*_ there are two possible cases for shared genes: the gene sets overlap or do not overlap.

#### Non-overlapping gene sets

First we calculated the expression summary *E*_*i*_ and *E*_*j*_ for pathways *i* and *j* respectively. Then, we estimated the pathway correlation as the Spearman correlation between the two pathway expression summaries

$$\begin{array}{c} \text{PathCor}\left(i,j\right)=\text{cor}\left(E_{i},E_{j}\right) \end{array}$$

#### Overlapping gene sets

Our approach to deal with overlapping pathway gene sets was to condition the correlation between the summaries for the pathways *G*_*i*_ and *G*_*j*_ on the summary for the genes common to both pathways (*G*_*i*∩*j*_ = *G*_*i*_ ∩ *G*_*j*_).

First, we calculated the summaries *E*_*i*_, *E*_*j*_, and *E*_*i*∩*j*_ corresponding to pathway *G*_*i*_, pathway *G*_*j*_ and the shared genes *G*_*i*∩*j*_. Then we estimated the partial correlation between the pathway summaries conditional on the summary for the shared genes

$$\begin{array}{c} \text{PathCor}\left(i,j\right)=\mathrm{cor}\left(E_{i},E_{j}|E_{i\cap j}\right) \end{array}$$

#### Hypothesis testing

We used a t-test to determine which experiment-level correlation coefficients were significantly different from 0.

$$\begin{array}{c} H_{0}:\text{PathCor}\left(i,j\right)=0\;H_{1}:\text{PathCor}\left(i,j\right)\neq 0 \end{array}$$

For the correlation coefficients between pathways without shared genes, the t-test is given by

$$\begin{array}{c} t=r\sqrt{\frac{n-2}{1-r^{2}}}∼t_{n-2} \end{array}$$

where *r* is the experiment-level correlation estimate.

For the correlation coefficients between pathways with shared genes, the t-test is given by

$$\begin{array}{c} t=r\sqrt{\frac{n-3}{1-r^{2}}}∼t_{n-3} \end{array}$$

where *r* is the experiment-level conditional correlation estimate.

### Meta-analysis estimates

#### Hunter-Schmidt estimator

We used the experiment-level correlation estimates to compute the overall correlation between two gene sets with a weighted average

$$\begin{array}{c} \bar{r}=\frac{\sum_{i=1}^{N}n_{i}r_{i}}{\sum_{i=1}^{N}n_{i}} \end{array}$$

where *n*_*i*_ is the number of samples for experiment *i*, *r*_*i*_ is the correlation estimate for experiment *i* and *N* is the total number of experiments [117].

#### Liptak p-value aggregation

Since we estimated the correlation coefficients at the experiment level, we first obtained a p-value from each of the experiments by testing if the experiment-level correlation was significant. In order to determine the significance of the overall correlation coefficient we combined the p-values from each experiment using Liptak’s method [121, 122]. The combined p-values across all experiments are given by

$$\begin{array}{c} p^{c}=1-\phi \left(Y\right) \end{array}$$

where

$$\begin{array}{c} Y=\frac{\sum_{i=1}^{N}n_{i}\Phi^{-1}\left(1-p_{i}\right)}{\sqrt{\sum_{i=1}^{N}n_{i}^{2}}} \end{array}$$

*ϕ* is the standard normal probability density function, Φ^−1^ is the standard normal inverse cumulative distribution function, *n*_*i*_ is the number of samples for experiment *i*, *p*_*i*_ is the p-value for experiment *i* and *N* is the total number of experiments.

After aggregating the experiment-level p-values for all pathway pairs, we adjust the combined p-values for multiple comparison using the Benjamini–Hochberg FDR method [123].

### Overlap coefficient

The overlap coefficient is a similarity measure for the overlap between two sets. For two sets *G* and *H*, the overlap coefficient is given by

$$\begin{array}{c} o_{GH}=\frac{\left|G\cap H\right|}{\min\{|G|,|H|\}} \end{array}$$

where 0 ≤ *o*_*GH*_ ≤ 1. The overlap coefficient is simply the size of the intersection divided by the size of the smaller of the two sets. We chose the overlap coefficient instead of other measures of overlap like the Jaccard index because it highlights whenever a pathway is a fully contained within another pathway. If a set *G* is a subset of *H*, the overlap coefficient is always 1. On the other hand, if the sets *G* and *H* are disjoint, the overlap coefficient is always 0.

### Ribosome gene sets

The annotation for the Ribosome pathway was retrieved from the KEGG REST server using the KEGGREST package (v. 1.10.1) [124]. We ran 1000 iterations for the no overlap and each overlap case using gene sets derived from the ribosome pathway annotation and random gene sets.

#### No overlap case

For the no overlap case, the KEGG Ribosome pathway was split in half. The ribosome pathway annotation, composed of 126 genes, was split into two non overlapping gene sets with 63 genes each with the following steps

1. Permute indexes of the genes belonging to the ribosome pathway
2. Split the gene set into two non overlapping gene sets *A* and *B*
3. Calculate the pathway summaries *E*_*A*_ and *E*_*B*_ for gene sets *A* and *B* respectively
4. Calculate the pathway correlation using the pathway summaries *E*_*A*_ and *E*_*B*_

For the random gene set, we sampled 126 genes present in the gene expression background, and split them with the following steps

1. Sample 126 genes from the background
2. Split the genes into two non overlapping gene sets *A*^*r*^ *B*^*r*^ with 63 genes each
3. Calculate the pathway summaries $E_{A}^{r}$ and $E_{B}^{r}$ for gene sets *A*^*r*^ and *B*^*r*^ respectively
4. Calculate the pathway correlation using the pathway summaries $E_{A}^{r}$ and $E_{B}^{r}$

#### Overlap cases

We created representative cases of gene overlap between two gene sets. In particular, we created two overlapping sets *s*_1_ and *s*_2_ from *n* distinct elements. In the first step, the two sets *s*_1_ and *s*_2_ share all but one element. In each consecutive step, we shift the indexes of one of the sets to decrease the number of shared elements between *s*_1_ and *s*_2_ until the last step when the two sets *s*_1_ and *s*_2_ do not have any elements in common.

$$\begin{array}{cc} \text{Step}\;1\;\; & s_{1}=\{1,\ldots ,\overset{⟵}{\overset{︷}{\left(n-1\right)}}\}\\ \;\;\; & s_{2}=\left\{1,\ldots ,\left(n-1\right),n\right\}\\ \text{Step}\;2\;\; & s_{1}=\left\{1,2,\ldots ,\left(n-1\right)\right\}\\ \;\;\; & \;s_{2}=\left\{\underset{\to }{2},\ldots ,\left(n-1\right),n\right\}\\ \text{Step}\;3\;\; & s_{1}=\left\{1,2,\ldots ,\overset{⟵}{\overset{︷}{\left(n-2\right)}}\right\}\\ \;\;\; & \;s_{2}=\left\{2,\ldots ,\left(n-2\right),\left(n-1\right),n\right\}\\ \text{Step}\;4\;\; & s_{1}=\left\{1,2,3,\ldots ,\left(n-2\right)\right\}\\ \;\;\; & \;\;s_{2}=\left\{\underset{\to }{3},\ldots ,\left(n-2\right),\left(n-1\right),n\right\}\\ & \vdots \\ \text{Step}\;n\;\; & s_{1}=\left\{1,\ldots ,\left(n-\left\lceil n/2\right\rceil \right)\right\}\\ \;\;\; & \;\;\;\;\;\;\;\;\;\;s_{2}=\left\{\left(n-\left\lceil n/2\right\rceil +1\right),\ldots ,n\right\} \end{array}$$

In order to consider different scenarios for the amount of shared genes between pathways, we built 9 different configurations of overlapping gene sets. These 9 overlap cases ranged from low overlap (*o*_*AB*_ = 0.0469) to high overlap (*o*_*AB*_ = 0.8532).

For the overlap cases, we split the KEGG Ribosome pathway was split into overlapping gene sets.

1. Permute indexes of the genes belonging to the ribosome pathway.
2. Split the gene set into two overlapping gene sets *A* and *B*.
3. Get the shared genes *A* ∩ *B* between sets *A* and *B*.
4. Calculate the pathway summaries *E*_*A*_, *E*_*B*_, and *E*_*A*∩*B*_.
5. Calculate the partial correlation between the summaries for the genes sets *A* and *B*, conditional on the shared genes *E*_*A*∩*B*_.

For the random gene sets, we sampled 126 genes present in the gene expression background and then split them into overlapping gene sets.

1. Sample 126 genes from the background.
2. Split the gene set into two overlapping gene sets *A*^*r*^ and *B*^*r*^.
3. Get the shared genes *A*^*r*^ ∩ *B*^*r*^ between the gene sets *A*^*r*^ and *B*^*r*^.
4. Calculate the pathway summaries *E*_*A*^*r*^_, *E*_*B*^*r*^_ and *E*_*A*^*r*^ ∩ *B*^*r*^_.
5. Calculate the partial correlation between the summaries for the genes sets *A*^*r*^ and *B*^*r*^, conditional on the shared genes *E*_*A*^*r*^ ∩ *B*^*r*^_.

#### ROC curves based on p-values

We generated a set of p-values based on the random gene sets and another set of p-values based on the ribosome gene sets. Assuming that a significant p-value for ribosome gene sets is a true positive while a significant p-value for random gene sets is a false positive, we assessed the ability of our method to identify truly significant correlation coefficients (Table 1). We used different p-value cut-offs for significance to build a receiver operating characteristic (ROC) curve.

**Table 1:** Confusion matrix for the ribosome and the random gene sets.

	Ribosome Gene Set	Random Gene Set
**Significant**	True Positive (TP)	False Positive (FP)
**Not significant**	False Negative (FN)	True Negative (TN)

Assignment of true positive (TP) and false positives (FP) based on the different p-value cut-offs fro significance from the ribosome and the random gene sets.

### Significant pathway overlap

We used Fisher’s exact test to identify significant overlaps between all pathway pairs. For pathway *i* with gene set *G*_*i*_ and pathway *j* with gene set *G*_*j*_, we used a contingency table based on their shared genes to perform an one-sided Fisher’s exact test (Table 2).

**Table 2:** Contigency table for shared genes between pathways.

	Genes in *G*_*i*_	Genes in $G_{i}^{c}$
**Genes in** *G*_*j*_	*G*_*i*_ ∩ *G*_*j*_	$G_{i}^{c}\cap G_{j}$
**Genes in** $G_{j}^{c}$	$G_{i}\cap G_{j}^{c}$	$G_{i}^{c}\cap G_{j}^{c}$

The contigency table splits the gene sets of pathways *i* and *j* into four disjoint sets. The shared genes between the two pathways, *G*_*i*_ ∩ *G*_*j*_, the genes unique to pathway *i*, $G_{i}\cap G_{j}^{c}$, the genes unique to pathway *j*, $G_{i}^{c}\cap G_{j}$, and the genes that do not belong to either pathway *i* or *j*, $G_{i}^{c}\cap G_{j}^{c}$.

Then we adjusted the corresponding p-values for multiple comparison using FDR, and considered an overlap significant if *p* < 0.05.

### PCxN webtool and bioconductor packages

The PCxN webtool is available at http://pcxn.org/. The webtool was built using open source software and libraries. The back-end of the website was developed using JSP(JavaServer Pages) powered by a Tomcat (http://tomcat.apache.org/, version 7.0.52) HTTP-server. MySQL (https://www.mysql.com/, version 5.5.46) was used to manage a relational database containing pathway correlation coefficients. The front-end user interface was developed using HTML and specialized libraries. The Jquery.js library (http://jquery.com/, version 2.1.1) was used to handle events. The canvasXpress.js library (https://canvasxpress.org/, version 13.5) was used to build heatmaps. The cytoscape.js library (http://js.cytoscape.org/, version 2.7.11) was used to build networks. PCxN is also available through Bioconductor as two distinct but interacting R packages. The pcxn package (http://bioconductor.org/packages/pcxn/) contains exploration and visualization wrapper functions that use data matrices stored in the pcxnData package (http://bioconductor.org/packages/pcxnData/).

## Supporting information

S1 Fig Venn diagrams for no overlap and overlap cases of the ribosome gene sets. (PDF)

S2 Fig Correlations estimates and ROC curves for the ribosome and the random gene sets. (PDF)

S1 Text Pathway summary statistic, impact of gene overlap (GO:BP), and robustness of the correlation estimates. (PDF)

S1 Table GSM and GSE accessions of gene expression data. (XLSX)

S2 Table Canonical pathways annotation. (XLSX)

S3 Table Gene overlap and correlation estimates for the canonical pathways in Fig 2B–2G. (XLSX)

S4 Table Alzheimer’s disease curated list. Domain expert curated list of genes associated with Alzheimer’s disease identified via genome wide association studies (GWAS).
(DOCX)

S5 Table Canonical pathways correlated with the Alzheimer’s disease curated list, and canonical pathways enriched for genes within the Alzheimer’s disease curated list. (XLSX)

S6 Table Genes associated with Alzheimer’s disease from the genetic association database. (XLSX)

S7 Table Canonical pathways enriched for genes associated with Alzheimer’s disease from the genetic association database. (XLSX)

S8 Table Results from gene set enrichment analysis on an Alzheimer’s disease profiling experiment. (XLSX)

S9 Table GEO accessions for the Alzheimer’s disease profiling experiment. (XLSX)

S10 Table Correlations between canonical pathways identified as enriched by gene set enrichment analysis and canonical pathways correlated with pathways identified as enriched by gene set enrichment analysis. (XLSX)
