# Supplementary material for: The Pathway Coexpression Network: Revealing pathway relationships
Source: PLoS Comput Biol. 2018 Mar 19;14(3):e1006042. doi: 10.1371/journal.pcbi.1006042 (PMC5875878; doi:10.1371/journal.pcbi.1006042)
Supplement: S1 Fig — (PDF) [file pcbi.1006042.s001.pdf]

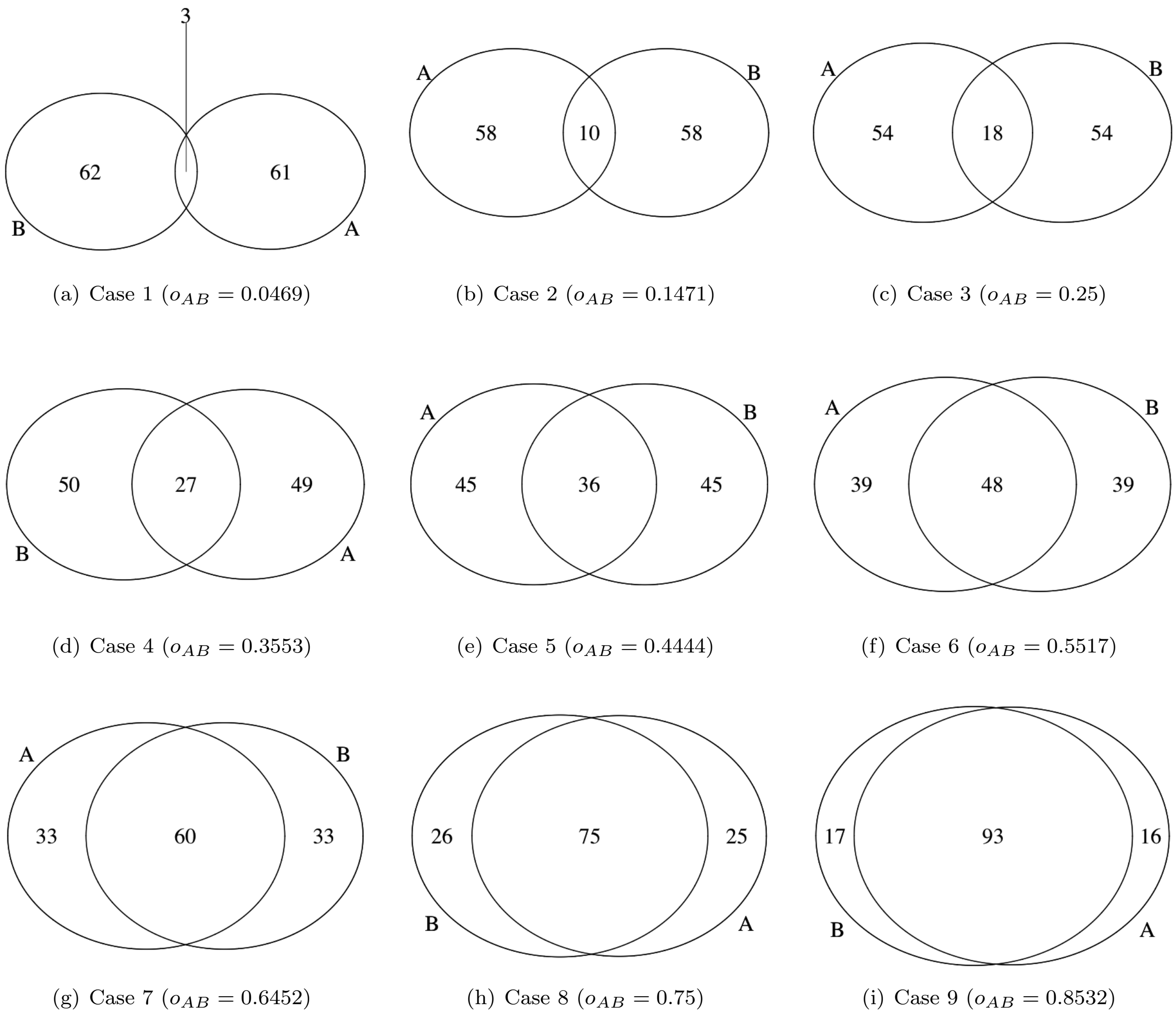

(a) Case 1 ($o_{AB} = 0.0469$) (b) Case 2 ($o_{AB} = 0.1471$) (c) Case 3 ($o_{AB} = 0.25$)

(d) Case 4 ($o_{AB} = 0.3553$) (e) Case 5 ($o_{AB} = 0.4444$) (f) Case 6 ($o_{AB} = 0.5517$)

(g) Case 7 ($o_{AB} = 0.6452$) (h) Case 8 ($o_{AB} = 0.75$) (i) Case 9 ($o_{AB} = 0.8532$)

Figure S1. Venn Diagrams for No Overlap and Overlap Cases of the Ribosome Gene Sets. Venn diagrams between the partitions used to separate the (KEGG) Ribosome pathway and random genes into gene sets with different degrees of shared genes.
