# Supplementary material for: The Pathway Coexpression Network: Revealing pathway relationships
Source: PLoS Comput Biol. 2018 Mar 19;14(3):e1006042. doi: 10.1371/journal.pcbi.1006042 (PMC5875878; doi:10.1371/journal.pcbi.1006042)
Supplement: S2 Fig — (PDF) [file pcbi.1006042.s002.pdf]

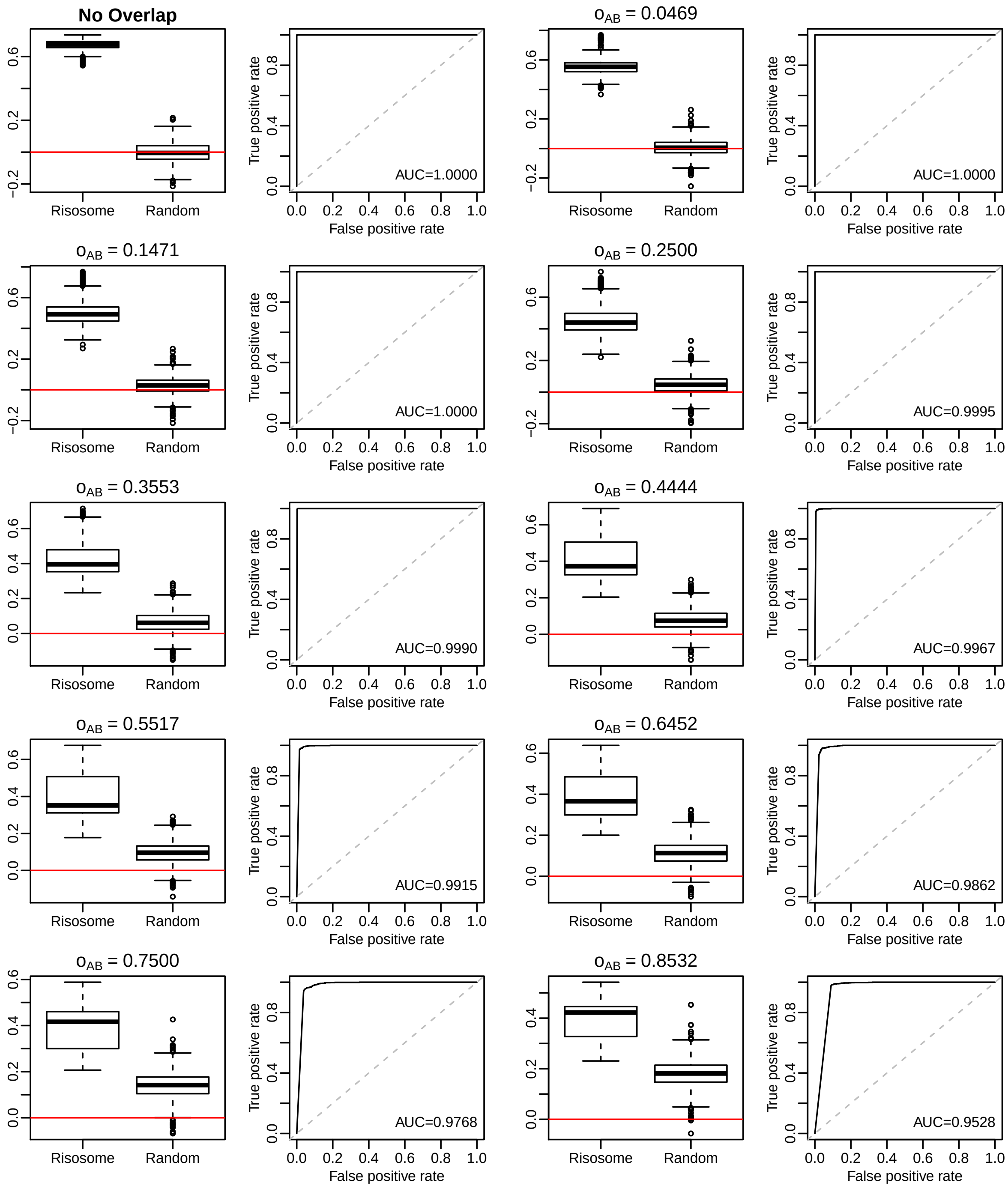


**Figure S2. Ribosome and the Random Gene Sets.** Boxplots of the correlation estimates between the ribosome gene sets and random gene sets under different cases of gene overlap. ROC curves for the ribosome gene sets and random gene sets under different cases of shared genes. We assume that a significant p-value ($p < 0.05$) for a ribosome gene set is a true positive, while a significant p-value for a random gene set is a false positive.
