# Supplementary material for: The Pathway Coexpression Network: Revealing pathway relationships
Source: PLoS Comput Biol. 2018 Mar 19;14(3):e1006042. doi: 10.1371/journal.pcbi.1006042 (PMC5875878; doi:10.1371/journal.pcbi.1006042)
Supplement: S1 Text — (PDF) [file pcbi.1006042.s003.pdf]

Supplementary Text 1

Pathway Summary Statistic

In PCxN we use the mean rank as the pathway summary statistic. We considered using the projection into the first principal component as the summary statistic. However, the variance explained by the first component was low in most of the curated experiments from normal human tissues. For each experiment from the curated collection, we estimated the percentage of variance explained by the first principal component for each of the 1,330 canonical pathways from the MSigDB CP v 5.1 collection. The barplot below shows the proportion of pathway for which the first principal component explains more than 50% of the variance.

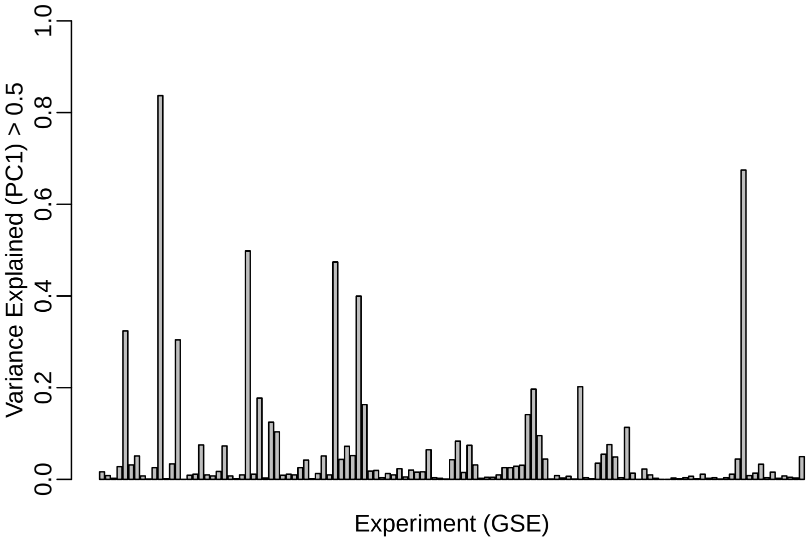

Figure 1: Proportion of canonical pathways for which the first principal component explains more than 50% of the variance across all experiments.

Impact of Gene Overlap (GO:BP)

We compared the number of significantly correlated pathways with the number of pathways which share a significant number of genes according to Fisher's exact test for the MSigDB GO:BP v 5.1 gene set collection.

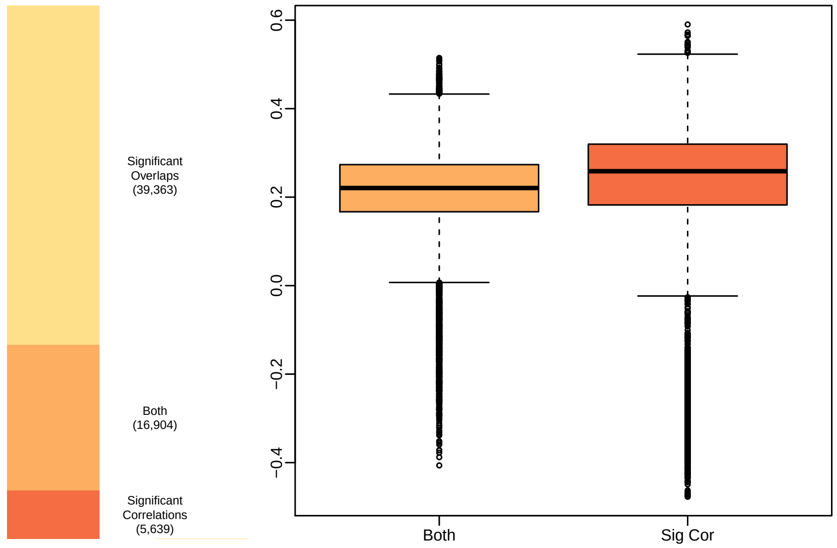

Figure 2: The stacked bar plot shows the number of pathways pairs with only significant correlations in red, with only significant overlaps in yellow, and with both in orange. The boxplots show the distribution of the correlation coefficients with pathway pairs with only significant correlations (red) and with both significant overlaps and significant correlations (orange).

The results for the GO:BP gene set collection are similar to the results that we reported for the Canonical Pathways in Figure 2D. We also observed more significant overlaps than significant correlations, and about 30% of the pathway pairs have both significant overlap and significant correlations. For pathway pairs with both significant overlaps and significant correlations, the correlations are lower on average that for pathway pairs with significant correlations only.

Robustness of the Correlation Estimates

We used Jackknife statistics to estimate the bias of the aggregated correlation estimates to assess the robustness of the coexpression network. The Jackknife statistics corresponding to the weighted average of the experiment-level correlation estimated leaving out one experiment at a time

$$\bar{r}_{(k)} = \frac{\sum_{i \neq k} n_i r_i}{n_i}$$

where r_i is the correlation estimate for experiment i, and n_i is the number of samples in experiment i.

The estimate for the bias is given by

$$\widehat{\text{Bias}} = (N-1)(\bar{r}_{(\cdot)} - \bar{r})$$

where N is the total number of experiments, $\bar{r}$ is the aggregated correlation estimate, and

$$\bar{r}_{(\cdot)} = \frac{1}{N} \sum_{k=1}^{N} \bar{r}_{(k)}$$

The estimate for the bias reflects the influence of each experiment i from the curated collection of normal human tissues on the aggregated correlation estimate $\bar{r}$.

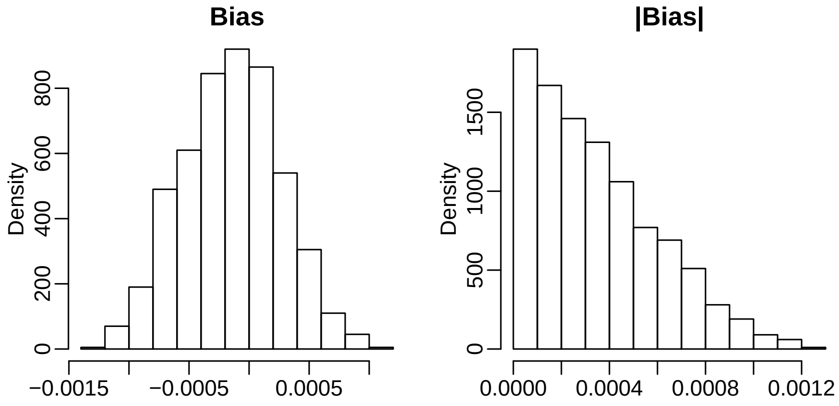

Figure 3: Histogram for the bias estimates (left), and the magnitude of the bias (right).

The overall bias is very low (mean -0.0001462, median -0.0001378, mean magnitude 0.0003480, median magnitude 0.0002962). The small bias of the correlation estimates of PCxN, demonstrate the robustness of the pathway coexpression network.
