# Supplementary material for: The Pathway Coexpression Network: Revealing pathway relationships
Source: PLoS Comput Biol. 2018 Mar 19;14(3):e1006042. doi: 10.1371/journal.pcbi.1006042 (PMC5875878; doi:10.1371/journal.pcbi.1006042)
Supplement: S4 Table — Domain expert curated list of genes associated with Alzheimer’s disease identified via genome wide association studies (GWAS). (DOCX) [file pcbi.1006042.s007.docx]

**Table S4. Alzheimer’s Disease Curated List.**

| **Support** | **Genes** |
| --- | --- |
| Early Onset Linked | APP, PSEN1, PSEN2 |
| FTLD GENES | MAPT, GRN, CHMP2B, VCP, C9orf72, FUS, TARDBP, CTNND2, PTN, HAVCR1, NYAP2, RNASEL |
| Late Onset Genome- Wide Associated Highly Suggestive | ZNF3, NDUFS3, MTCH2, IGHV1-67, TP53INP1, ACE, ATXN1, HLA-DRA, HLA-DRB4, HLA-DQ-A1, HLA-DQB, HLA-DQB1, HLA-DQA1, DPYSL2, AX747894, RIN3, LGMN, GOLGA5, HS3ST1, SQSTM1, TREML2, NDUFAF6, ECHDC3, AP2A2, ADAMST20, IGH, SPPL2A, TRIP4, SCIMP |
| Late Onset Genome-Wide Significant | APOE, CD33, BIN1, PTK2B, CLU, ABCA7, CR1, PICALM, MS4A6A, MS4A4E, CD2AP, SORL1, SLC24A4, DSG2, INPP5D, MEF2C, NME8, ZCWPW1, CELF1, FERMT2, CASS4, ADAM10, TREM2, HLA-DRB5, HLA-DRB1 |

Domain expert curated list of genes associated with Alzheimer’s disease identified via genome wide association studies (GWAS).
